# Objective Improvement in Cognitive Function following Aortic Coarctation Repair in an Adult

**DOI:** 10.1055/s-0041-1724004

**Published:** 2021-10-04

**Authors:** Danielle Hayes, Bryce French, Swee Lian Tan

**Affiliations:** 1 Department of General Surgery, Swedish Medical Center, Seattle, Washington; 2 Department of Vascular Surgery, Virginia Mason, Seattle, Washington

**Keywords:** aortic coarctation, cognitive impairment, Montreal cognitive assessment

## Abstract

Coarctation of the aorta is a rare finding in adults and can present with vague symptoms. We report a case of a 64-year-old cognitively impaired female who presented with fatigue and tinnitus. After extensive workup, she was diagnosed with coarctation of the aorta and subsequently underwent repair. After operative intervention for her coarctation, her cognitive impairment was found to have an objective improvement evidenced by the Montreal Cognitive Assessment.

## Introduction

Coarctation of the aorta, the sixth most common form of congenital heart defect, rarely presents in the adult population. Pathologically, it is a narrowing in the lumen of the aorta secondary to medial wall thickening and aortic wall tissue infolding. 1 When presenting in adulthood, the most common symptoms are hypertension and fatigue. Failure to intervene may lead to congestive heart failure, aortic dissection or rupture, and endocarditis or intracranial bleeding. 1 2 We report an interesting case where the diagnosis and treatment of coarctation of the aorta in an adult led to objective and subjective improvement in her cognitive function.

## Case Presentation

A 64-year-old female with history of hypertension, depression, and fatigue presented with balance impairment and swishing in her ears. She was known to have baseline cognitive impairment confirmed by the Montreal Cognitive Assessment (MoCA) score of 19 determined by her primary care provider.

**Fig. 1:**
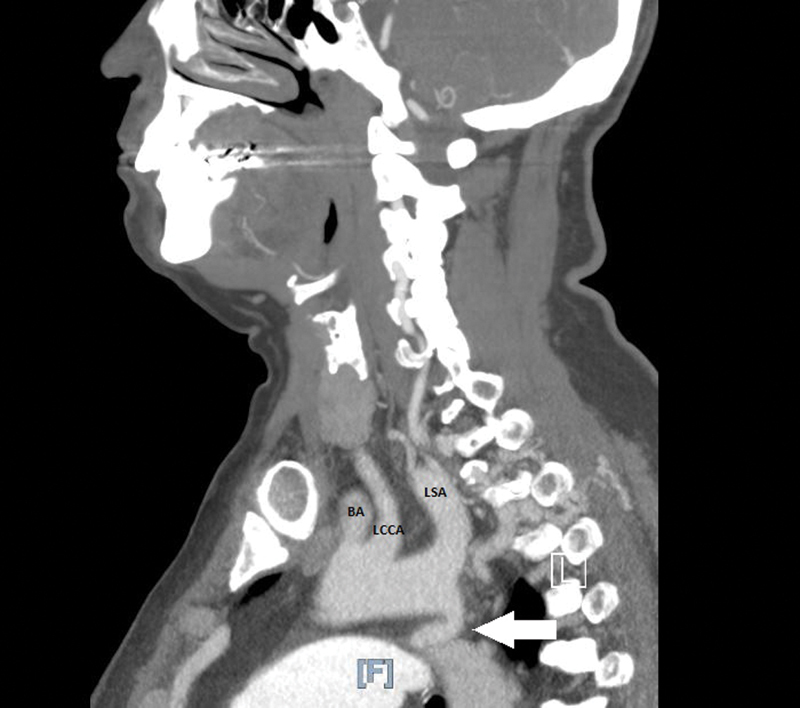
Computed tomography angiography notable for aortic coarctation with narrowing of aortic lumen distal to origin of left subclavian artery. White arrow pointing at site of coarctation. BA, brachiocephalic artery; LCCA, left common carotid artery; LSA, left subclavian artery.

Due to concern for vertebral artery disease, she underwent an extracranial cerebrovascular duplex investigation, which demonstrated bilateral vertebral artery stenosis of 50 to 90%. This prompted further evaluation with computed tomography angiogram that demonstrated severe narrowing of the proximal descending thoracic aorta, immediately distal to the origin of the left subclavian artery ( Fig. 1 ). An aortogram and selective left subclavian artery angiogram was obtained for further evaluation and noted no significant stenosis of the vertebral arteries but a very high-grade stenosis of the aorta just distal to the subclavian artery consistent with coarctation of the aorta. On further questioning, the patient revealed she had reproducible leg pain caused by exercise and alleviated by rest consistent with claudication.

A left axillary bifemoral bypass using 8-mm ringed polytetrafluoroethylene was performed. At the 2-week follow-up appointment, she stated that she was able to walk without leg pain, her balance issues had resolved, and the “swishing” in her ears had improved.

Her primary care provider readministered the MoCA screen and the patient scored a 26, within normal range. Subjectively her energy, sleep, and memory had all improved. Objectively, her hypertension improved, and she required only one of her three antihypertensive medications postoperatively.

## Discussion

Aortic coarctation presenting in adults is rare and can be difficult to diagnose due to the nonspecific symptoms. 1 2 3 These symptoms are secondary to the juxtaductal aortic narrowing resulting in increased left ventricular afterload and hypertension proximal to the obstruction with decreased flow distal to the obstruction. 1 Dyspnea, fatigue, headache, and hypertension are the most common presenting signs reported in the literature. 1 2 3 Physical examination findings consisting of decreased or absence femoral pulses can aid in the diagnosis.

To our knowledge, this is the first case of coarctation of the aorta demonstrated to show an objective improvement in cognitive function following surgical treatment. The patient's MoCA score improved from a level of moderate cognitive impairment to within the normal range 14 weeks after her operation.

Our first hypothesis is that her low MoCA score was secondary to chronic fatigue. A meta-analysis by Cockshell and Mathias 4 lends some credence to this theory, as they noted objective cognitive deficits in patients with chronic fatigue syndrome in the domains of attention, memory, and reaction time. In our patient, the cognitive deficits seen may be due to the physiology of the coarctation syndrome rather than the symptom of fatigue.

A second hypothesis is that the patient's chronic hypertension (requiring three antihypertensive medications) resulted in an alteration of cerebral microcirculation. It is known that hypertension has detrimental effects on cognition and that malignant hypertension impacts cerebrovascular autoregulation. 5 The alteration in cerebral microcirculation can lead to regional cerebral perfusion deficits causing a suppression of brain activity and cognitive dysfunction. 6 Her cognitive improvement could therefore be secondary to improvement in cerebral microcirculation from decreased episodes of hypertension and reduced afterload from her left axillary artery to bifemoral arteries bypass.

A third hypothesis is that coarctation of the aorta leads to altered cerebral blood flow possibly impairing cognition. A study performed by Wong et al 7 utilized transcranial Doppler to assess patients with coarctation of the aorta and demonstrated increased intracranial vessel stiffness, impaired vasoreactivity to carbon dioxide, widened pulse pressure, and a trend toward impaired vasodilator response to visual stimuli. These alterations in cerebral blood flow may cause impaired endothelial function resulting in a reduced increase in perfusion during neuronal activation which could contribute to poor cognitive performance. 7

There are multiple described surgical approaches to coarctation, including open resection and interposition graft, stenting, or extra-anatomic bypass. During our angiogram, we were unable to traverse a wire across the coarctation prohibiting placement of stent. Due to the requirement of a thoracotomy for interposition graft placement, the patient elected to undergo a left axillary artery to bifemoral arteries bypass, despite the lower rates of patency. Her graft has remained patent with most recent follow-up after 3 years.

Coarctation of the aorta is a challenging diagnosis to make in adults without a high index of suspicion or imaging demonstrating this pathology. We have identified cognitive impairment as an additional symptom of coarctation of the aorta that can be used to help identify this rare but serious condition. However, we are reporting a single case with only one set of pre- and postprocedure cognitive assessments, and the conclusions must be guarded.
